# *Escherichia coli* as a sentinel in the assessment of antimicrobial resistance in the tilapia production chain: from production environment to the final product

**DOI:** 10.3389/frabi.2024.1461662

**Published:** 2024-10-22

**Authors:** Sthéfany Da Cunha Dias, Letícia Roberta Martins Costa, Ana Beatriz Garcez Buiatte, Marcus Vinícius Coutinho Cossi, Luís Augusto Nero, Ricardo Seiti Yamatogi, Luciano dos Santos Bersot, Juliano Gonçalves Pereira

**Affiliations:** ^1^ Department of Animal Production and Preventive Veterinary Medicine, São Paulo State University "Júlio de Mesquita Filho" (UNESP), Botucatu, São Paulo, Brazil; ^2^ Faculty of Veterinary Medicine, Federal University of Uberlândia (UFU), Uberlândia, Minas Gerais, Brazil; ^3^ Department of Veterinary Medicine, Federal University of Viçosa (UFV), Viçosa, Brazil; ^4^ Department of Veterinary Sciences, Federal University of Paraná (UFPR), Palotina, Brazil

**Keywords:** antibiotics, *E. coli*, microbial sensitivity, tilapia, resistance

## Abstract

**Introduction:**

The intensification of tilapia production has increased animal density in tanks, leading to more frequent exposure to pathogenic agents and compromising the quality of fish products. Antimicrobial resistance is a global concern that affects human treatment, and sentinel microorganisms like *Escherichia coli* are crucial for monitoring production chains, especially in aquaculture, where research is still limited. The aim of this study was to identify the presence of *E. coli* and investigate its antimicrobial resistance profiles throughout the entire tilapia production chain.

**Methods:**

A total of 240 samples were collected from various points in the production process: carcasses before scaling (Ca), scaling wastewater (Sw), filleting wastewater (Fw), fillet washing wastewater (Tw), fillet handling surfaces (Su), and pre-packaged fillets (Pf). The samples were collected during 10 visits, each corresponding to animals from different farms. *E. coli* isolates were identified using MacConkey agar and biochemical tests. Phenotypic resistance profiles were determined using nine classes of antimicrobials. Extended- spectrum b-lactamase (ESBL) production was identified with ceftazidime and cefotaxime and confirmed by a double-disc synergy test. Isolates were classified as sensitive or resistant based on the inhibition zone. Multidrug-resistant (MDR) was defined as resistance to at least one agent in three or more antimicrobial categories, while extensively drug-resistant (XDR) was defined as resistance to at least one agent in all but two or fewer categories.

**Results:**

Overall, 50.8% of the samples (122/240) tested positive for *E. coli*, with 403 isolates identified. Of these, 33% (133/403) were resistant to at least two antimicrobials, and 20% (48/240) of the samples had MDR isolates, with the highest frequency found at the filleting point (Fw), which also had the only XDR profile. Resistance was most commonly observed against amoxicillin (35.73%), tetracycline (30.77%), and ciprofloxacin (26.30%).

**Discussion:**

These findings emphasize the importance of *E. coli* as an indicator of antimicrobial resistance throughout tilapia processing and highlight the need for good production practices and qualified technical support to mitigate risks to public health, animal health, and the environment.

## Introduction

Brazil is recognized worldwide for its agro-export potential, moving the country’s economy and generating employment and income in the various work chains involved in agribusiness. Contributing to food security in several regions of the world, fish represents an important source of animal protein (Food and Agriculture Organization of the United Nations, 2020). The production of this food matrix is a growing market, with fish farming reaching 860,355 tons produced in 2022 in Brazil (Pedrini et al., 2023). This highlights the importance and growth of this chain, and its economic and social impacts since about 8% of the world population is dependent on this sector (Food and Agriculture Organization of the United Nations, 2020).

The production of tilapia (*Oreochromis* spp.) stands out worldwide. In Brazil, tilapia represents about 63.93% of the Brazilian fish production, keeping the country as the fourth largest producer in the world (Pedrini et al., 2023). The intensification of the system is a direct reflection of globalization, which has a growing demand for animal products (Dawood and Koshio, 2016). As a result, the adoption of biosecurity measures and good agricultural practices are essential for the prevention of disease outbreaks that compromise animal health and even consumer health due to their zoonotic nature (Van Boeckel et al., 2017).

Thus, the increased use of antimicrobials has been a common practice in intensive tilapia production systems, which are used as treatment, prophylaxis or metaphylaxis of batches (Smith, 2020). These practices have been pointed out as one of those responsible for the emergence of multidrug-resistant microorganisms, being a risk to animal, human and environmental health (Cabello, 2006). Considering that most of the Brazilian fish farms are in net cages in hydroelectric reservoir (Moura et al., 2016; Camargo and Amorim, 2020), the environmental impact becomes more relevant, as it increases the risk of multidrug-resistant bacteria dissemination. Therefore, these considerations highlight the importance of the topic within the concept of one health (Van Boeckel et al., 2017).

Among the measures that can be adopted, the investigation and monitoring of the antimicrobial resistance profile of bacteria is one of the first necessary actions (Preena et al., 2020; Caputo et al., 2023). This allows the elaboration of specific policies to combat resistance within aquaculture (Preena et al., 2020). In this sense, the use of sentinel microorganisms, as *E. coli*, has been shown to be effective and reliable (Mencía-Ares et al., 2022). In this context, *E. coli* is useful because it is characterized as an important disseminator of resistance genes (Poirel et al., 2018). This reinforces its importance as a target microorganism in monitoring programs (Lihan et al., 2021).

The rise in multidrug resistance globally poses a serious public health threat. Several recent studies have reported the emergence of MDR bacterial pathogens from diverse sources, emphasizing the necessity for the proper use of antibiotics. Furthermore, the routine implementation of antimicrobial susceptibility testing is crucial to identify effective antibiotics and detect emerging MDR strains (Algammal et al., 2022; Ibrahim et al., 2024; Mabrok et al., 2024).

Zoonotic pathogens such as *E. coli*, *Salmonella* spp., and *Staphylococcus* spp. can be present in fish production systems, compromising the safety of animal-derived products and affecting consumer health (Dhama et al., 2013). *E. coli* is known for its resistance to many antibiotics and its ability to spread resistance genes, highlighting the risk of contaminated water to biosafety and human health (Ng et al., 2018; Preena et al., 2021).

Although *E. coli* generally does not cause disease in fish, it can express virulence factors and cause infections in humans, making the implementation of good manufacturing practices and self-monitoring programs in production systems essential (Greenlees et al., 1998). As a gram-negative rod from the *Enterobacteriaceae* family, *E. coli* serves as an indicator of hygiene and can be transmitted through food, water, and soil (Croxen et al., 2013; Jang et al., 2017). Besides commensal strains, pathogenic strains that cause approximately 2 million deaths annually exist and can be classified into seven groups based on their virulence mechanisms (Croxen et al., 2013). *E. coli* is frequently associated with foodborne outbreaks, especially with raw products, emphasizing the need for an integrated approach to assess epidemiological impacts and risks (Walker et al., 2018).

Despite this importance, there are few studies that assess the antimicrobials resistance of bacteria in the fish production chain. Thus, the objective of this work was to evaluate the presence and resistance profile of *E. coli* from tilapia production chain.

## Materials and methods

### Sample collection

The project was carried out in a tilapia-processing industry that works under Official Brazilian Inspection System of Animal Products, located in southwestern Brazil. Ten visits to the industry were carried out, with a single origin of tilapia being slaughtered and collected at each visit. Fish-farming were identified from A to F (all under intensive production systems) and samples were collected at the following production points: carcasses before scaling (Ca); scaling wastewater (Sw); filleting wastewater (Fw); fillet toilet wastewater (Tw); fillet handling surface (Su); and pre-packaged fillets (Pf) (**Table 1**). For logistical reasons, this study considered Ca and Sw as representative of the Fish-farming microbiological conditions.

**Table 1:** Description of points, samples, and collection methods carried out in the tilapia-processing industry.

Kind of sample	Collect point*	Method	Area or volume sample	n/visit	n total
Fish-farming conditions	Ca	Rinse	100 mL	10	100
Fish-farming conditions	Sw	Flasks	25 mL	1	10
Industrial process	Fw	Flasks	25 mL	1	10
Industrial process	Tw	Flasks	25 mL	1	10
Industrial process	Su	Swabbing	400 cm^2^	1	10
Industrial process	Pf	Rinse	100 mL	10	100
**Total**				24	240

*Ca, carcass; Sw, scaling wastewater; Fw, filleting wastewater; Tw, fillet toilet wastewater; Su, fillet handling surface; Pf, pre-packaged fillets.

Ca and Pf points were sampled by superficial rinsing in sterile bags containing 100 mL of sterile saline solution (0.85% w/v). Su point was sample by swabbing two sterile sponges previously moistened with 20 mL of saline solution (0.85% w/v). For this procedure, sterile molds measuring 100 cm^2^ (10 cm x 10 cm) were used to delimit the area to be sampled, which was collected in 4 different places, totaling 400 cm^2^. At the points where the water was collected (Sw, Fw and Tw), sterile flasks containing sodium thiosulfate were used. After collection, the samples were placed in styrofoam box and kept at 4 °C until microbiological analysis.

### Isolation and characterization of *E. coli*

Samples were subjected to *E. coli* isolation according to American Guidelines Public Health Association (APHA, 2001) Under sterile conditions, except for Su, 25 mL of each sample were added to 225 mL of Buffered Peptone Water (BPW CM0509 – Oxoid, Thermo Fisher Scientific, Waltham – EUA) (1:10). For Su, 20 mL were added to 180 mL of BPW (1:10). All samples were than homogenized in a stomacher for 60 seconds and incubated at 37°C for 18–24h. After this period, they were incubated in *Escherichia coli* broth (EC Broth CM0979 – Oxoid, Thermo Fisher Scientific, Waltham – EUA) at 45°C for 48h. Subsequently, aliquots of the broth were streaked onto MacConkey Agar (CM 0115, Oxoid, Thermo Fisher Scientific, Waltham – EUA) and incubated at 37°C for 18–24 h. If present, a total of four lactose fermenting colonies with typical *E. coli* morphology (on MacConkey agar, the colonies are pink, typically smooth, and may have a shiny appearance) and one non-lactose fermenting colony (colonies appear yellow or colorless) were selected.

All the suspected colonies selected were submitted to biochemical identification using EPM (Escola Paulista de Medicina), Mili (Motility, Indole, and Lysine) (De Toledo, 1982), and Simmons citrate (CM 0155, Oxoid, Thermo Fisher Scientific, Waltham – EUA). In typical biochemical tests for *Escherichia coli*, the following results are often observed: in the EPM test, gas production and glucose fermentation; and in the MiLLi test, positive results for lysine, indole, and motility, and negative for citrate. It is important to note that some *Escherichia coli* pathotypes may show negative results for lysine decarboxylation and motility.

Samples biochemically confirmed as *E. coli* were stored on Nutrient agar (CM 0003, Oxoid, Thermo Fisher Scientific, Waltham – EUA) and in Brain Heart Infusion (CM 1136, Oxoid, Thermo Fisher Scientific, Waltham – EUA) broth added with 10% glycerol and kept frozen.

### Characterization of the phenotypic profile of antibiotic resistance in *E. coli* isolates

The sensitivity of *E. coli* isolates to antimicrobial agents was evaluated using the Kirby-Bauer disk diffusion methodology (Baurer et al., 1966), according to international recommendations (Clinical and Laboratory Standards Institute, 2018; Clinical and Laboratory Standards Institute, 2020). A total of nine classes of antimicrobials, commonly used in animal production and for human health, were tested: amoxicillin – AMO (10 μg); ceftiofur – CTF (30 µg); aztreonam – ATM (30 µg); imipenem – IPM (10 µg); ciprofloxacin – CIP (5 µg); tetracycline – TET (30 µg); gentamicin – GEN (10 μg), sulfamethoxazole + trimethoprim – SUT (23.75/1.25 μg), chloramphenicol – CLO (30 μg) and azithromycin – AZI (15 μg) (Antimicrobial disks, Interlab, São Paulo, Brazil). Extended-spectrum β-lactamase production (ESBL) two antimicrobials were used as screening, ceftazidime – CAZ (10 μg) and cefotaxime-CTX (5 μg) (Antimicrobial disks, Interlab, São Paulo, Brazil), both third generation cephalosporins (W. Therapeutics Guideline Group, 2020). In addition, the isolates were characterized by their resistance to extended-spectrum b-lactamases (ESBL) by a double-disc synergy test (EUCAST, 2013). The results were classified according to the Clinical and Laboratory Standards Institute (2020), as sensitive (S), intermediate (I), or resistant (R) (Clinical and Laboratory Standards Institute, 2020).

The *E. coli* isolates were categorized as sensitive or resistant based on the inhibition zone. Isolates resistant to three or more classes of antimicrobials were classified as multidrug-resistant (MDR), which is defined as resistance to at least one agent in three or more antimicrobial categories. Extensively drug-resistant (XDR) was defined as resistance to at least one agent in all but two or fewer categories, while pandrug-resistant (PDR) was defined as resistance to all agents in all categories (Magiorakos et al., 2012) The positive reference standard strain used to ensure the accuracy and reliability of the tests was *Escherichia coli* ATCC 25922 (Biomedh, Minas Gerais, Brazil).

### Statistical analysis

Descriptive statistics were used to characterize the frequency of *E. coli* and resistant isolates at each point. *E. coli* frequency results between the initial (Ca) and final point of process (Pf) were compared by chi-square. The chi-square test was also used to compare the MDR *E. coli* frequencies between fish-farms, for that, samples that presented at least one positive MDR isolate were considered positive. For all analysis the GraphPad Prisma 9.2.0 software was used (P<0.05). The figures were constructed using the RStudio (RStudio: Integrated Development Environment for R, 2024) packages ggplot2 (Chang et al., 2016) and UpSetR (Conway et al., 2017).

## Results

A total of 50.8% of samples (122/240) were positive for *E. coli*. Despite the 31-percentage-point reduction in the frequency of *E. coli* between the initial (Ca) and final (Pf) stages of tilapia processing industry, all analyzed points had positive samples. Among the positive samples, 403 isolates were identified as *E. coli* by using biochemical tests (**Table 2**).

**Table 2:** Frequency of *Escherichia coli* and number of isolates from a tilapia processing unit in southwestern Brazil.

Sample points*	n	Positive samples (%)	*E. coli* isolates (n)
Fish-farming conditions			
Ca	100	66 (66,0)	221
Sw	10	5 (50,0)	20
Industrial process			
Fw	10	6 (60,0)	20
Tw	10	7 (70,0)	19
Su	10	3 (30,0)	11
Pf	100	35 (35,0)	112
**Total**	240	122 (50,8)	403

*Ca, carcass before scaling; Sw, scaling wastewater; Fw, filleting wastewater; Tw, fillet toilet wastewater; Su, fillet handling surface; Pf, pre-packaged fillets.

The antimicrobial susceptibility test showed that 36% (145/403) of the *E. coli* strains were susceptible to all tested antimicrobials and 33% (133/403) were resistant to at least two antimicrobials (**Supplementary Table S1**). The highest frequencies of resistance presented by the isolates were against amoxicillin (35.73%), tetracycline (30.77%) and ciprofloxacin (26.30%), respectively. It was observed that the *E. coli* was associated with lower resistance to gentamicin (1.99%), azithromycin (2.73%), and ceftiofur (2.98%), aztreonam (4.,71%) and imipenem (6.95%) (**Table 3**).

**Table 3:** Antimicrobial resistance of *Escherichia coli* obtained at different stages of a tilapia-processing industry located in southwestern Brazil.

Class	Antibiotic	Resistance of isolates (n=403)	
		n	(%)
Aminoglycosides	Gentamicin	8	1,99
Beta-lactams	Amoxicilin	144	35,73
	Aztreonam	19	4,71
Carbapenems	Imipenem	28	6,95
Cephalosporins	Ceftiofur	12	2,98
Amphenicols	Chloramphenicol	47	11,66
Fluorquinolones	Ciprofloxacin	106	26,3
Macrolides	Azithromycin	11	2,73
Sulfonamides	Trimethoprim-sulfamethoxazole	50	12,41
Tetracyclines	Tetracycline	124	30,77

**Figure 1:**
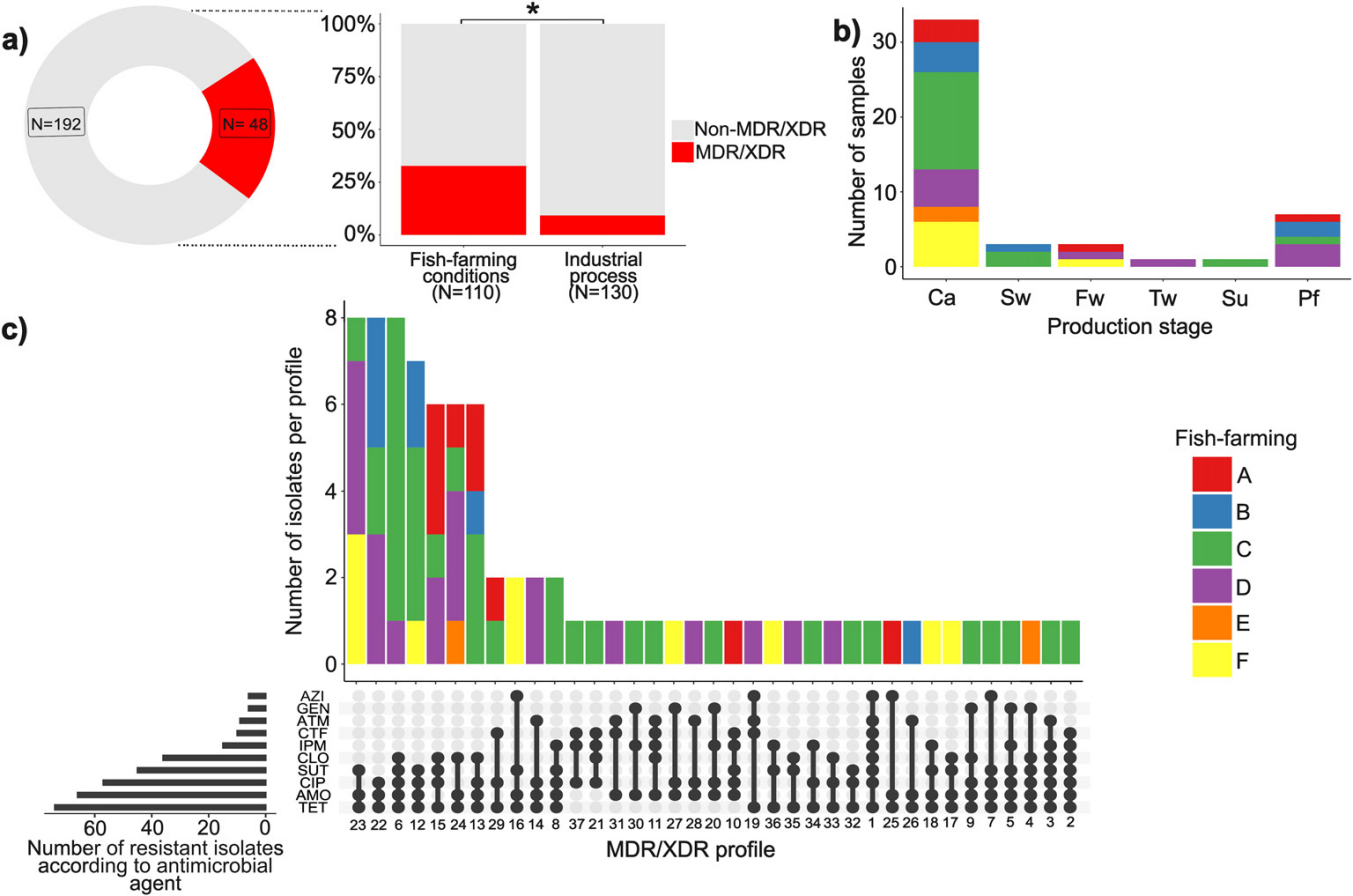
Multidrug resistance (MDR) and Extensively drug-resistant (XDR) of *Escherichia coli* obtained at different stages of a tilapia-processing industry located in southwesternBrazil. **(A)** Number of MDR/XDR samples by production stage. *Comparison between fish-farming conditions and industrial process using chi-square test (p<0.05). **(B)** Distribution of MDR/XDR samples according to the different production stages. Ca: carcass. Sw: scaling wastewater. Fw, filleting wastewater; Tw, fillet toilet wastewater; Su, fillet handling surface; Pf, pre-packaged fillets. **(C)** Distribution of isolates according to the 36 MDR and 1 XDR profiles identified. TETTetracycline; CIP-Ciprofloxacin; SUT-Sulfamethoxazole and trimethoprim; CLOChloramphenicol; AZI-Azithromycin; CTF-Ceftiofur; IPM-Imipenem; ATM-Aztreonam; AMOAmoxicillin; GEN-Gentamycin.

Considering the results of resistance to the antimicrobials tested, it was possible to identify 20.0% (48/240) of samples with at least one MDR *E. coli* isolate (**Figure 1A**). The highest frequency of positive samples was obtained from representative points of the Fish-farming microbiological conditions (Ca and Sw) (chi-square test, p=0.0038, **Figure 1A**). However, this difference was only identified by the results of fish-farming C, which had 41.66% (15/36) of the MDR positive samples obtained from Ca and Sw points (**Figure 1B**). It is worth mentioning that these MDR positive samples were identified in two different visits to the slaughterhouse, five samples from a first batch and nine from a second one, showing a pattern of results from this fish-farming.

Analyzing the resistance results presented by each isolate, 62 profiles were identified (**Supplementary Table S1**). Of these, 58,06% (36/62) were considered MDR isolate profiles, ranging from 3 to 8 antimicrobial classes and 1.61% (1/62) was classified as an XDR isolate profile, exhibiting resistance to antimicrobials from 8 different classes (**Figure 1C**) and no samples were identified as PDR. Eleven resistance profiles were formed by five or more classes of antimicrobials, nine of which were exclusive or contained isolates from fish-farming C. Eleven profiles brought together more than one isolate, and eight had isolates from more than one fish-farming. Among the MDR resistance profiles, 36.14% (30/83) isolates showed simultaneous resistance to TET-CIP-SUT, 46.99% (39/83) showed resistance to AMO-TET-CIP, and 40.97% (34/83) showed resistance to TET-AMO-SUT.

## Discussion

Considering all the collection points, *E. coli* was identified in a prevalence of 50.8%. A similar result was obtained by Dewi et al. (2022) in Malaysia, where 44.5% of tilapia samples were positive. Some studies indicate that *E. coli* does not occur naturally in the microbiota of fish, which is influenced by the habitat (Guzmán et al., 2004), and thus this pathogen is transferred to these animals by the environment and during handling. The reduction of positivity samples for *E. coli* between the initial (Ca) and the final collection point (Pf) shows that, despite the presence of the agent, the industry’s self-control programs have been efficient in reducing biological hazards. In this context, it is important to emphasize the role of the government as a supervisory agent and the industry as responsible for seeking a safety product for the consumer (Lupien, 2007; De Filippis et al., 2018; Preena et al., 2020). On the other hand, the presence of *E. coli* at all analysis points reinforces its important role as a sentinel microorganism for monitoring the resistance of microorganisms to antimicrobials (Mencía-Ares et al., 2022).

It is important to emphasize that antimicrobial resistance (AMR) refers to the ability of microorganisms to remain alive and active even in the presence of antimicrobial agents. These agents include antibiotics, disinfectants, and food preservatives, which are used to control microbial growth, inhibit their multiplication, or even eliminate them. Antimicrobials can be classified as natural, semi-synthetic, or synthetic and operate through various mechanisms, causing significant impacts on the microorganisms’ metabolic and physiological processes. For instance, β-lactams and glycopeptides affect cell wall synthesis, while macrolides and tetracyclines block protein production. Other antimicrobials, such as sulfonamides, interfere with metabolic pathways, and fluoroquinolones affect DNA replication and translation (Tenover, 2006; Varela et al., 2021).

In this context, the present study observed that while more than a third of the *E. coli* isolates were susceptible to all the antimicrobials tested, another third exhibited resistance to at least two drugs, thus being classified as multidrug-resistant (Jeong et al., 2007). Similar studies have demonstrated antimicrobial resistance in *E. coli* from fish. In Brazil, Rocha et al. (2014) analyzed 44 *E. coli* strains isolated from tilapias collected in markets, which showed low resistance to sulfamethoxazole-trimethoprim (4.54%) and tetracyclines (15.9%), with no isolates resistant to gentamicin, imipenem, or ciprofloxacin. In a study conducted in Malaysia, *E. coli* strains were isolated from tilapias within the production chain, with the authors finding 42.7% of isolates to be multidrug-resistant (MDR) (Dewi et al., 2022).

Resistance to tetracyclines (31.2%), chloramphenicol (12.7%), gentamicin (5.1%), and ceftiofur (0.0%) was similar to our findings, whereas resistance to ciprofloxacin (15.3%) was lower. In Bangladesh, Amin et al. (2024) conducted a study on 500 fish samples (tilapias and pangas) collected from the market, where the levels of resistance to chloramphenicol (7.0%) were similar to our findings, although resistance to ciprofloxacin (15.0%) was lower, and resistance to tetracyclines (40.0%), aztreonam (38.0%), gentamicin (9.0%), and sulfamethoxazole (38.0%) was higher (Amin et al., 2024). The levels of resistance in *E. coli* within fish production in African countries were analyzed in a meta-analysis by Moffo et al. (2024), which observed a high prevalence of MDR strains (43.1%) on the continent. Resistance to tetracyclines (66.4%), gentamicin (18.0%), and chloramphenicol (44.4%) was greater than what was observed in our study, while resistance to ciprofloxacin (15.1%) was lower (Moffo et al., 2024).

In addition to these data, studies are also concerned about the resistance against amoxicillin and tetracycline, which is a risk for the environment and future generations due to its widespread misuse (Weir et al., 2012). Resistance to tetracycline is considered frequent in most aquatic productions due to its wide use and corroborates the findings in the present study (Tuševljak et al., 2013). Conversely, the sensitivity of *E. coli* isolates to gentamicin and aztreonam, observed in our study, can be explained by the less common use of these drugs in aquaculture (Rocha et al., 2014).

Another important result was the *E. coli* isolates resistance to Chloraphenicol. This pharmacological base has been banned in animal production since 2003, because its residues constitute a risk to public health (MAPA, 2003). Despite this, an antimicrobial from the same class, florfenicol, is used to treat fish diseases, as it is an effective antimicrobial against a broad spectrum of pathogens (Botelho et al., 2015; Preena et al., 2020). The role of horizontal gene transfer in the dissemination of resistance should be considered as a possible explanation for our finding (Richardson et al., 2018) since some mechanisms of resistance to florfenicol also provide resistance to chloramphenicol (Schwarz et al., 2004; Pacheco-Silva et al., 2014). However, the literature suggests that this drug is used even after its legal ban (Pacheco-Silva et al., 2014). Therefore, the illegal use of this drug inducing resistance in the analyzed microbiome should be considered (Smith and Lewin, 1993; Miller and Harbottle, 2018).

Antimicrobial-resistant bacteria are currently one of the greatest challenges in human and veterinary medicine. In aquaculture, they are associated with the presence of residues in the aquatic environment and alterations in the local microbiome, contaminating fish and increasing the risk of resistant pathogens reaching humans. A major issue is that the limitations of therapies and the efficacy of pharmacological treatments are insufficient to combat resistance acquired by pathogenic microorganisms (Heuer et al., 2009).

Throughout their evolution, microorganisms have developed various mechanisms to resist the effects of antimicrobial agents, involving complex molecular and cellular systems. It is important to note that resistance to a single agent often leads to the development of resistance to multiple drugs in new variants. Consequently, multidrug-resistant bacteria can significantly compromise the effectiveness of treatments (Wright, 2011; Christaki et al., 2020).

Antibiotic resistance in bacteria can be classified into three types: intrinsic, acquired, and adaptive. Intrinsic resistance is linked to the natural characteristics of bacteria that make them inherently resistant to certain antibiotics. Acquired resistance, on the other hand, occurs when previously sensitive bacteria develop resistance due to genetic mutations or the incorporation of external genetic material via horizontal gene transfer (Holmes et al., 2016).

This process can occur through three main mechanisms: in transformation, bacteria absorb free DNA from the environment or under laboratory conditions and integrate it into their genome; in transduction, bacteriophages transfer DNA, including resistance genes, between bacteria during replication, spreading resistance; and in conjugation, resistance genes are transferred directly between bacteria through physical contact, aided by transferosomes and coupling proteins (Holmes et al., 2016; Munita and Arias, 2016).

Adaptive resistance, in contrast, arises in response to specific environmental signals such as stress or nutrient conditions. Unlike intrinsic and acquired resistances, which are permanent, adaptive resistance is temporary and reverts to the original state once the stimulus is removed. This type of resistance results from changes in gene expression, mediated by epigenetic modifications like DNA methylation, and involves the regulation of efflux pumps and porins (Salimiyan Rizi et al., 2018; Lee, 2019). Moreover, multidrug-resistant bacteria can transfer their resistance genes to other species in various environments, such as hospitals, the food industry, the human intestinal tract, and agriculture (Varela et al., 2021).

As occur in other parts of the world (Machowska and Stålsby Lundborg, 2018; Garcia et al., 2020), in Brazil it is relatively easy to purchase antimicrobials for use in animals, often without a veterinarian’s prescription (Garcia et al., 2020). Despite the existence of laws that state which drugs can be used, improvements still need to be done to curb this practice. Legislations that regulate the commercialization of drugs for veterinary use need to be created and improved, and adequate oversight needs to be implemented.

The higher frequency of MDR isolates observed at sample points related to fish-farming microbiological conditions reinforces this concern about the misuse of antimicrobials. This corroborates the fact that the indiscriminate use of antimicrobials in animal production is a concern and requires immediate changes, reinforcing international recommendations on surveillance and monitoring programs (Smith et al., 2013; Preena et al., 2020).

The higher consumption of antimicrobials is normally associated with intensive production systems, a condition that brings economic benefits but also increases the possibility of disease in tilapia (Jackson et al., 2020; Wencewicz, 2019). This occurs because the intensification of these systems exposes the animal to more stressors, weakening its immunological barriers. Knowing this reality, many producers carry out prophylaxis or metaphylaxis of their batch of fish, increasing the chance of developing multidrug-resistant bacteria (Wencewicz, 2019; Rigos et al., 2021). Despite this, it should be known that good practices in production and qualified technical support are ways to achieve an intensive production system with low consumption of antimicrobials (Rigos et al., 2021). These variations in the practices of each production system may explain the high frequency of MDR *E. coli* observed in fish-farming C, compared to other farms.

In addition to animal health problems, other challenges related to MDR bacteria are the risks of reaching humans through the food chain, and the impacts they may have on the environment (Islam et al., 2019). This requires a broad approach that directs the antibiotic use, with adequate and assisted indication in all links of the animal production chain (Manyi-Loh et al., 2018; de Alcântara Rodrigues et al., 2020).

The wide variation in resistance profiles found in our study reveals the variety of antimicrobials that may be used in the tilapia production. Furthermore, when animals are submitted to a challenge, their microbiome and the aquatic environment microbiome can cause the differences observed between isolates (Ho et al., 2000). The highest resistance profiles were found in *E. coli* isolated at sample points related to animal production, showing that the primary production is the bottleneck in tilapia production chain in relation to bacterial resistance.

The present study did not identify any extended-spectrum beta-lactamase-producing strain, unlike previous studies such as the one by Sivaraman et al. (2020). One possibility for this difference is the characteristic of local production exerting less selective pressure on the microbiome. Furthermore, the absence of the enzyme production phenotype does not rule out the possibility of strains presenting the gene and transmitting it to other bacteria present in the medium and, consequently, the risk to public health (Kazemian et al., 2019). However, a molecular analysis to answer this gap was not performed in this research.

The damage done by years of indiscriminate use of antibiotics cannot be undone, but alternatives already exist that can minimize their use. The use of herbal medicines appears as a natural alternative, non-aggressive to the environment and with antimicrobial properties (Valladão et al., 2015). Another strategy is the use of essential oils in the prevention and treatment of diseases in fish, contributing to the reduction of the use of antibiotics (Cunha et al., 2018). The use of vaccines reduces the use of antimicrobials and contributes to animal health in intensive production (Håstein et al., 2005). All these alternatives must also include good agricultural practices, water management, proper cleaning, proper disease diagnosis and improvement in infrastructure. Finally, probiotics are also an alternative to the use of antimicrobials, as they influence water quality, increase the immune response and antiviral effects (Balcazar et al., 2006).

## Conclusion

The presence of *E. coli* in all stages of tilapia processing reinforces its importance as a sentinel microorganism for resistance surveillance. Furthermore, the high frequency of multidrug resistance isolates, especially in samples related to the microbiota of the fish-farming. The study warns about the risk to public health, animal health and the environment, reinforcing the importance of good practices in animal production and qualified technical support.

## Data Availability

The raw data supporting the conclusions of this article will be made available by the authors, without undue reservation.
